# Cupping Therapy for Migraine: A PRISMA-Compliant Systematic Review and Meta-Analysis of Randomized Controlled Trials

**DOI:** 10.1155/2021/7582581

**Published:** 2021-03-24

**Authors:** Jihye Seo, Hongmin Chu, Cheol-Hyun Kim, Kang-Keyng Sung, Sangkwan Lee

**Affiliations:** ^1^Department of Oriental Gynecology, Se-Myung University Korean Medicine Hospital, Republic of Korea; ^2^Department of Internal Medicine and Neuroscience, College of Korean Medicine, Wonkwang University, Iksan, Republic of Korea; ^3^Department of Internal Medicine, Wonkwang University Gwangju Korean Medicine Hospital, Gwangju, Republic of Korea; ^4^Internal Medicine and Neuroscience, Jangheung Integrative Medical Hospital, Wonkwang University, Iksan, Republic of Korea

## Abstract

**Background:**

Migraine is a common reason for primary headache disorders. Cupping is a frequently used traditional intervention for controlling pain including migraine. There have been no systematic reviews on the clinical effects of cupping on migraine.

**Objective:**

This systematic review and meta-analysis aimed to evaluate the effectiveness of cupping therapy for migraine. The search strategy was built for the presence of related keywords, such as “migraine” and “cupping therapy”, in the title and abstract of research articles indexed in the MEDLINE, EMBASE, CENTRAL, and other databases. The randomized controlled trials (RCTs) of cupping therapy for migraine were searched and selected from inception to May 2019. We searched eight databases including PubMed, EMBASE, Cochrane Central Register of Controlled Trials. The selection process and the quality assessment were performed by 2 authors independently. The meta-analysis was conducted and qualitative analysis was also performed.

**Results:**

218 studies were identified, and 6 RCTs were enrolled in this review. In comparison to drugs, wet cupping showed a higher total effective rate (TER). In the dry cupping plus acupuncture, the result of TER showed more effectiveness (RR 1.05, 95% CI 0.99 to 1.12, *P*=0.13) compared with acupuncture alone, but there was no statistically significant difference. In qualitative analysis, the results showed wet cupping plus drugs treatment could quickly relieve pain and significantly improve patients' quality of life and wet cupping could reduce headache pain.

**Conclusion:**

Cupping therapy could be effective for the treatment of migraine. However, the qualities of the evidence were low, so well-designed RCTs are needed to confirm the effectiveness of cupping. Systematic review registration: PROSPERO registration number is CRD42017054979.

## 1. Introduction

Migraine, a common primary headache, is characterized by attacks of pulsating moderate-to-severe headaches that can be associated with nausea and/or photophobia or phonophobia [1]. Migraine causing the disability of life and hindering social relationships is the second disease of the most burdensome neurological disease worldwide [2, 3]. Therefore, appropriate diagnosis and first-line acute and prophylactic treatments have been constantly required to relieve this burden.

Although pharmacologic treatment guidelines for migraine headaches have been developed and updated, there are limitations in drug therapy such as side effects and overuse [4–6]. Therefore, many kinds of migraine treatments including acupuncture, herbal medicine, moxibustion, blood-letting, and cupping are used clinically in East Asia as complementary and alternative medicine [7, 8].

**Figure 1:**
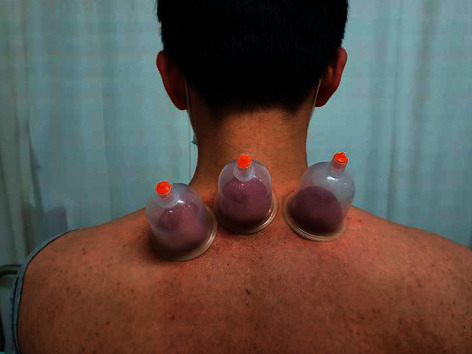
Standard dry cupping therapy.

Cupping is an East Asian medicine therapy that has been used for thousands of years [9]. The physician puts special cups on the patient's skin for cupping therapy and creates local suction as shown in Figure 1. Various types of cupping have been practiced in clinical fields including retained cupping, wet cupping, moving cupping. Cupping therapy is also widely practiced in Middle East Asia and is also known as Hijima therapy [10]. In spite of its use worldwide, only some systematic reviews of cupping therapies have been published [11–15]. Several case reports and randomized clinical studies show the efficacy of cupping for blood circulation disorders and nervous system diseases [16, 17]. Especially for treating headache and migraine, cupping therapies have been practiced [18]. However, there has been no systematic review on the effectiveness of cupping therapy on migraine. Therefore, it seems pertinent to evaluate the effectiveness of cupping for treating migraine. In this systematic review, cupping for treating migraine is evaluated and summarized.

## 2. Methods and Analysis

We developed the protocol of this study ourselves. The protocol is registered on the International Prospective Register of Systematic Reviews (PROSPERO) (registration number: CRD42017054979) and was published in 2017. We conducted this study according to the protocol [19].

### 2.1. Data Sources and Search Methods

The following electronic databases were searched from their inception to 12 May 2019: MEDLINE, EMBASE, and the Cochrane Central Register of Controlled Trials (CENTRAL). We searched MEDLINE using the PubMed database. We also searched four Korean medical databases (OASIS, the Korean Traditional Knowledge Portal, the Korean Medical Database, and DBPIA) and the China National Knowledge Infrastructure (CNKI). The search strategy was developed using the terms about migraine and cupping therapy. The actual search terms were modified and used according to each database's environment. The used search strategies are listed separately in the supplemental section (Supplementary 1).

### 2.2. Study Selection

The selection process was independently conducted by two authors using the Endnote referencing software. In the primary selection, we reviewed the titles and abstracts and selected the studies likely to be suitable for our study. Then, the full texts of primary selected studies were reviewed and confirmed. When there was a discrepancy in the selection, the final selection was made after sufficient discussion. We used the following inclusion and exclusion criteria.

### 2.3. Inclusion Criteria

Inclusion criteria were as follows: (1) the prospective randomized controlled clinical trials (RCTs) or quasi-randomized controlled trials (quasi-RCTs) that were written in English, Chinese, or Korean; (2) studies with participants diagnosed with migraine; (3) studies that used cupping treatment alone or as an adjunct to other treatments (if the control group received the same treatment as the experimental group) as experimental interventions; and (4) studies that used a placebo or conventional drug therapy as control interventions.

### 2.4. Exclusion Criteria

Exclusion criteria were as follows: (1) the studies that included cupping treatment in both groups as the intervention; (2) the full text could not be available or impossible to extract data; and (3) a thesis or dissertation.

### 2.5. Data Extraction and Quality Assessment

Two independent authors performed the data extraction and quality assessment of the RCTs using a data extraction form using Excel software. The form was designed by all the authors in consensus. It included the year of study publication, characteristics of participants, sample size, intervention details, outcomes, and adverse events. Quality evaluation of each study was assessed with the “Risk of bias” tool from the Cochrane Handbook Version 6.0, which includes random sequence generation, allocation concealment, blinding of the participants and personnel, blinding of the outcome assessments, incomplete outcome data, selective reporting, and other sources of bias [20]. The results of this process cross-checked by two authors and disagreements were resolved with a discussion.

### 2.6. Data Analysis

#### 2.6.1. Outcome Measures

The primary outcomes were measured by changes in headache pain intensity and improved effectiveness such as the total treatment effective rate (TER). Secondary outcomes were evaluated from the following: (1) migraine-related symptoms as measured with validated questionnaires; (2) migraine-accompanying symptoms; and (3) adverse events.

#### 2.6.2. Data Synthesis

The data synthesis was performed with the Cochrane Collaboration's software program Review Manager (RevMan) V.5.3 for Windows. Quantitative synthesis was conducted dividing the included studies according to the types of interventions. For dichotomous data, TER, we presented the outcomes as relative risks (RRs) with 95% CIs. For continuous data such as headache pain intensity and migraine-associated symptoms, we calculated the effect size of the interventions using the mean differences (MDs) with 95% CIs. If each study presented similar outcome values on different scales, we used the standard mean difference (SMD) with 95% CIs. We calculated the data for the meta-analysis using the random effects model. The outcomes that were insufficient to quantitative synthesis were described qualitatively. When meta-analysis was conducted, the heterogeneity between the outcomes was assessed by the *I*^2^ statistic value calculated with the RevMan program. The *I*^2^ more than 50 was considered to indicate high heterogeneity. Subgroup analysis was attempted to find the cause of high heterogeneity. To conduct subgroup analyses, we explored the types of control drugs, whether patients have traditional syndrome differentiation, and treatment duration in each study. If the number of studies available for the subgroup is insufficient, we described the probable cause of the heterogeneity.

The publication bias was not assessed because the number of studies in each meta-analysis group was insufficient.

The results of this study were assessed with the Grading of Recommendations Assessment, Development and Evaluation (GRADE) to assess the strength of the evidence [21].

## 3. Results

### 3.1. Results of the Search and Description of the Included Studies

**Figure 2:**
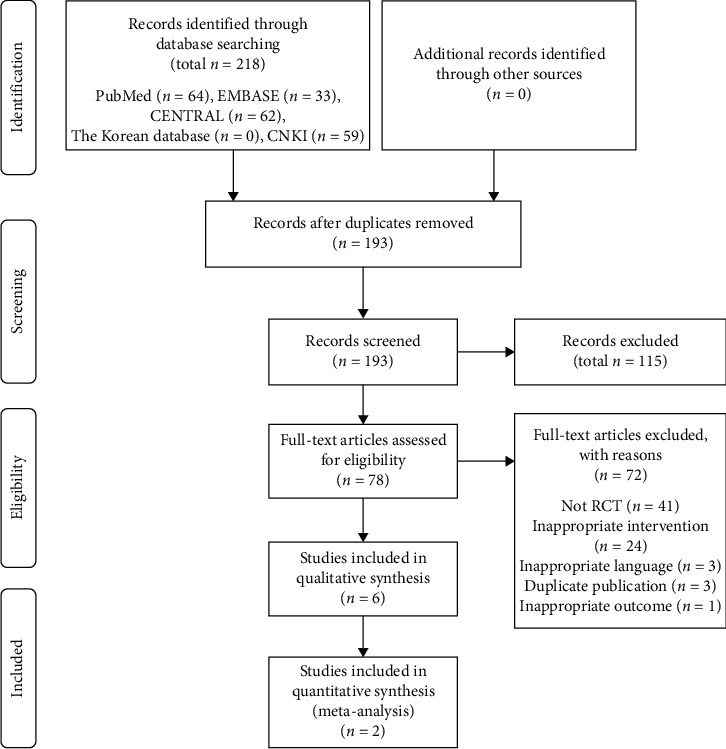
The flow chart of the study selection process. CENTRAL: Cochrane Central Register of Controlled Trials, CNKI: Chinese National Knowledge Infrastructure, and RCT: randomized controlled trial.

A total of 218 articles were searched in 8 electronic databases. Through the selection process, 6 RCTs were selected. The selection process and reasons for exclusion are presented in Figure 2. A total number of 510 patients participated and the sample size ranged from 60 to 130. The included participants were diagnosed with migraine. 6 RCTs [22–27] were conducted in China. For data analysis, the included studies were divided according to experiment and control interventions. We divided the studies into three groups: (1) wet cupping versus drugs (3 RCTs) [22, 23, 27]; (2) dry cupping accompanied with acupuncture versus without acupuncture (2 RCTs) [24, 25]; and (3) wet cupping accompanied with drugs versus drugs (1 RCT) [26]. We conducted a quantitative analysis in group 2 and a qualitative analysis in groups 1 and 3. The details of the included studies are shown in Table 1. Four of the six studies used wet cupping while two studies used dry cupping. In the three studies, only cupping was performed alone, all of them were wet cupping, and in the remaining three studies, acupuncture or drug was accompanied by cupping. The frequency of cupping therapy and duration of treatments varied. The number of cupping points was one to eight and the most frequent cupping point was EX-HN5. The characteristics of experiment intervention are presented in Table 2.

The outcome evaluations were TER, headache intensity (HI), migraine attack frequency (MF), migraine attack intensity (MI), headache duration time (HD), migraine-accompanying symptoms (MAS), migraine score, the number of days the headache has completely disappeared (ND), and 24-hour migraine quality of life questionnaire (24hMQOLQ).

All RCTs used the TER as the outcome. The degree of treatment effectiveness was generally divided into four categories or three categories. TER is the rate of participants with an improvement of the migraine symptoms among the total participants. 4 RCTs [23, 25–27] measured the HI using the visual analogue scale (VAS). MF, MI, HD, and MAS were measured by a scoring system in 1 RCT [27]. Migraine score was the score of HI, MF, HD, and MAS. It was measured by 1 RCT [23]. ND was reported in Li's study [24], and 24hMQOLQ in Zhang's study [26]. Data on adverse events (AEs) were reported in 2 RCTs [23, 27].

### 3.2. Risk of Bias in the Included Studies

**Figure 3:**
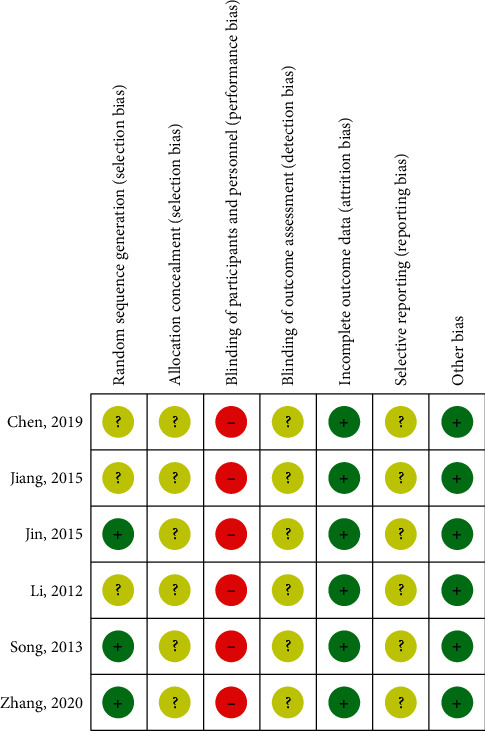
Summary of the risk of bias.

The result of the risk of bias is shown in Figure 3. In the evaluation of random sequence generation, 3 RCTs [23, 25, 26] [28] had reported adequate random sequence generation, while random sequence generation was unclear in 3 RCTs [22, 24, 27]. In the evaluation of allocation concealment, all RCTs did not report the allocation concealment. In the included RCTs, no RCT applied placebo or sham intervention as a control. For that reason, the participants could not be blinded and bias of blinding of participants and personnel was evaluated high in all RCTs. The blinding of outcome assessment was unclear in all RCTs. In all studies, the results of enrolled participants without dropouts were analyzed. Thus, the items of incomplete outcome data were evaluated low. In the evaluation of selective reporting, all included studies without the protocol were evaluated unclear.

### 3.3. Effects of Cupping Therapy

#### 3.3.1. Wet Cupping versus Drugs

Three studies [22, 23, 27] (210 participants) compared wet cupping versus drugs (flunarizine, ibuprofen, or diclofenac sodium). The designs of the three studies were different, and there were also differences in the control treatment and the number of treatments. Therefore, the studies were described qualitatively.

Jiang's study [14] (60 participants) compared wet cupping versus drugs (flunarizine). It reported the results of TER. In this study, it was found that the effects of TER in wet cupping were higher than that in drugs. The differences were statistical significance (*P* < 0.05).

Song's study [15] (90 participants) compared wet cupping versus drugs (flunarizine and add ibuprofen at acute attack). It reported the results of TER, HI using the VAS and the migraine score. In this study, it was found that the effects of TER in wet cupping were higher than that in drugs. The differences were statistical significance (*P* < 0.05). And both HI and the migraine scores in wet cupping improved significantly better than those in drugs.

Chen's study [19] (60 participants) compared wet cupping versus drugs (diclofenac sodium at acute attack). It reported the results of TER, HI using the VAS MF, MI, HD, and MAS. In this study, it was found that the effects of TER in wet cupping were higher than that in drugs. The differences were statistical significance (*P* < 0.05). And the rest of the outcomes in wet cupping improved significantly better than those in drugs.

Two of the three studies reported data on adverse events (AEs). Song's study [23] reported no AEs in wet cupping, 34 AEs (32 cases of fatigue and 2 cases of epigastric pain) in drugs. Chen's study [27] reported 2 AEs (2 cases of dizziness) in wet cupping, 9 AEs (1 case of dizziness, 4 cases of epigastric pain, 2 cases of vomiting, and 2 cases of nausea) in drugs. RR of AEs was 0.07 (95% CI 0.00 to 1.68) in the wet cupping group.

#### 3.3.2. Dry Cupping plus Acupuncture versus Acupuncture

**Figure 4:**
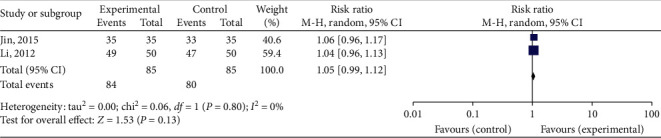
Forest plot of dry cupping plus acupuncture versus acupuncture, outcome: TER. TER: total effective rate.

Two studies [24, 25] (170 participants) compared dry cupping accompanied with acupuncture versus without acupuncture. These studies reported TER. The result of the meta-analysis in TER showed that dry cupping with acupuncture was more effective in TER than acupuncture alone (RR 1.05, 95% CI 0.99 to 1.12, *P*=0.13). The statistic *I*^2^ showed no heterogeneity (*I*^2^ = 0%). However, there was no statistically significant difference in TER (Figure 4).

Jin's study [25] reported the results of HI using the VAS. In this study, the HI in dry cupping plus acupuncture was 2.29 (MD –2.29, 95% CI: −2.62, −1.96) lower than in acupuncture alone.

Li's study [24] reported the results of ND. In this study, it was found that dry cupping plus acupuncture significantly reduced ND (MD –1.98, 95% CI: −2.91, −1.05) compared with acupuncture alone (*P* < 0.05).

In this group, no studies were reported on AEs.

#### 3.3.3. Wet Cupping plus Drugs versus Drugs

In this group, 1 RCT [26] was included. The study was described qualitatively.

Zhang's study [26] (130 participants) compared wet cupping accompanied with drugs (rizatriptan benzoate) against drugs (rizatriptan benzoate). It reported the results of TER, HI using VAS and 24hMQOLQ. In this study, it was found that the effects of TER, HI and 24hMQOLQ in wet cupping plus drugs were higher than that in drugs. The differences were statistical significance (*P* < 0.05). These results suggest that wet cupping plus drugs treatment can quickly relieve pain and significantly improve patients' quality of life. Data on AEs was not reported.

### 3.4. Publication Bias

Publication bias was not measured because there were insufficient studies included in each meta-analysis group.

### 3.5. Quality of the Evidence

The quality of evidence was assessed in the wet cupping versus drugs group and dry cupping plus acupuncture versus acupuncture group using GRADE criteria. The levels of evidence were low for TER, HI, and migraine score in wet cupping compared to drugs. We downgraded the level of evidence due to the risk of bias and imprecision. In dry cupping plus acupuncture compared to acupuncture, the levels of evidence were low for HI, TER, and ND due to the risk of bias and imprecision. The results of the evidence assessment and each reason for a decreasing level are shown in Table 3.

## 4. Discussion

Cupping therapy as traditional medicine has potential benefits for several types of pain, hypertension, herpes zoster, and cough [9]. According to the previous RCT, cupping therapy also has the potential benefit for migraine [22, 27]. We conducted this systematic review to show whether cupping therapy is efficacious for migraine. We analyzed 6 RCTs with 510 migraine participants in this review and the results show that wet cupping and dry cupping could improve the migraine symptoms. No significant AEs were identified in the studies included in this review.

Meta-analysis was performed by dividing the groups according to the type of intervention. Dry cupping accompanied with acupuncture could improve the TER (RR 1.05, 95% CI 0.99 to 1.12, *P*=0.13) compared to acupuncture alone; the difference was not statistically significant. Nevertheless, it was found that the effects of HI and ND in dry cupping accompanied with acupuncture were higher than those in acupuncture alone in each included study. In clinical practice, cupping and acupuncture could be applied together to reduce the intensity and duration of a headache in migraine patients. However, this meta-analysis could not confirm a significant difference due to the small number of studies. Although it is convenient to use acupuncture and cupping together in clinical practice, it is unclear whether there is any benefit in cost-effectiveness.

Due to the different control treatment and the insufficient number of studies, the studies were analyzed qualitatively in the wet cupping versus drugs group and wet cupping plus drugs versus drugs. The results of each study showed that wet cupping was more effective in the reduction of HI than drugs. Wet cupping was effective in MF, MI, HD, and MAS, which are secondary outcomes. But quantitative synthesis was impossible because of inconsistent outcome measurements. Similarly, the results showed that wet cupping accompanied by drugs significantly improved symptoms of migraine. Although there were a few reports of adverse events among the included studies, it was possible to suggest that the cupping therapy was a safe treatment because the adverse reaction was less than drugs in included studies.

This review is the first comprehensive systematic review and meta-analysis about cupping therapy for migraine. In addition, the levels of evidence were presented through assessment of the quality of the evidence. The quality of the level of evidence was low in the wet cupping versus drugs group and dry cupping plus acupuncture versus acupuncture group. The number of studies was insufficient, and various limitations were observed for the included studies, so referring to these points at the time of further studies would help to increase the evidence. The findings of our review might provide evidence-based information on the therapeutic effects of cupping therapy to support treatment decisions on migraine.

There are some limitations of this systematic review. The number of RCTs included was insufficient and the sample sizes were small. Meta-analysis was performed on TERs, but there was a possibility of bias due to different evaluation criteria of TERs. In addition, the included RCTs did not follow the CONSORT reporting guideline, so the quality of the study was low. These limitations were responsible for reducing the level of evidence. According to the ICHD-3, there are two main subtypes of migraine: migraine with aura and migraine without aura. It is important to distinguish a migraine with or without aura in migraine attacks in clinical trials, but there was no included RCT that differentiated between migraine with or without aura. We could not analyze it distinguishing between migraine with or without aura.

Regardless of the potential bias and limitations, all the included studies concluded that cupping therapy had beneficial therapeutic effects in treating migraine. The clinical practitioners may consider choosing cupping therapy for nonpharmacological migraine therapy, based on their own clinical experience and environment. To have cupping therapy be accepted by the evidence-based medicine, well-designed with powered sample size RCTs will be required. In addition, to confirm quantitative results for HI, MF, MI, and HD, the use of these evaluations should be considered when performing RCT in the future.

## 5. Conclusion

Cupping therapy has potential therapeutic effects on treating migraine. Further larger and rigorously designed RCTs are needed to confirm the cupping therapy's therapeutic effect. Additional economic studies might be considered for future studies to compare the cost-effectiveness of cupping therapy plus acupuncture or cupping therapy plus drugs.

## Figures and Tables

**Table 1:** Details of the included studies.

Study ID	Sample size (randomized/analyzed)	Participant age (years)	Duration of disease (years)	The differentiated patterns	(A) Treatment group intervention (n)	(B) Control group intervention (n)	Treatment duration/Follow-up duration	Main outcomes	Adverse events
Chen, 2019	(A) 30/30 (B) 30/30	(A) 40.62 ± 3.55 (B) 42.45 ± 3.64	(A) 2.34 ± 0.25 (B) 2.62 ± 0.57	None	Wet cupping	Diclofenac sodium	3 months/none	1. TER 2. Migraine attack frequency score 3. Migraine attack intensity score 4. Migraine attack intensity score 5. Headache duration time score 6. Headache intensity (VAS)	(A) 2 (dizziness 2) (B) 9 (dizziness 1, abdominal pain 4, vomiting 2, nausea 2)
Jiang, 2015	(A) 30/30 (B) 30/30	(A)20–65 (B) 18–57	(A) 0.5–16 (B) 2–13	None	Wet cupping	Flunarizine	4 weeks/none	1. TER	NR
Song, 2013	(A) 45/45 (B) 45/45	(A) 35.4 ± 3.1 (B) 36.1 ± 2.3	(A) 4.3 ± 2.2 (B) 3.5 ± 2.8	Cold coagulation and blood stasis	Wet cupping	Flunarizine plus Ibuprofen (at acute attack)	8 weeks/none	1. TER 2. Headache intensity (VAS) 3. Migraine score	(A) 0 (B) 34 (fatigue 32, abdominal pain 2)
Jin, 2015	(A) 35/35 (B) 35/35	(A) 34 ± NR (B) 33 ± NR	(A) 7.24 ± NR (B) 6.81 ± NR	None	Dry cupping plus acupuncture	Acupuncture	8 weeks/none	1. TER 2. Headache intensity (VAS)	NR
Li, 2012	(A) 50/50 (B) 50/50	(A) 36.5 ± NR (B)31.2 ± NR	(A) 9.2 ± NR (B)8.7 ± NR	None	Dry cupping plus acupuncture	Acupuncture	15 days/none	1. TER 2. The number of days the headache has completely disappeared	NR
Zhang, 2020	(A) 65/65 (B) 65/65	18–45	>1	None	Rizatriptan benzoate plus wet cupping	Rizatriptan benzoate	20 days	1. TER 2. 24hMQOLQ 3. Headache intensity (VAS)	NR

24hMQOLQ: 24-hour migraine quality of life questionnaire, NR: not reported, TER: total treatment effective rate, and VAS  visual analogue scale.

**Table 2:** Characteristics of experiment intervention of included studies.

Study ID	Methods of cupping	Number of cupping points	Cupping points	Other intervention	Number of treatment sessions/duration	Frequency of sessions/time of cupping
Chen, 2019	Wet cupping	1-2	EX-HN5	None	Unclear/3 months	Unclear (at the time of migraine attack)/unclear
Jiang, 2015	Wet cupping	2	BL17	None	8/4 weeks	2 per week/20 minutes
Song, 2013	Wet cupping	8	Mei hua point (梅花穴)1), Xiang ling point (項棱穴)2), EX-HN5, GB20, GV14	None	16/8 weeks	2 per week/15 minutes
Jin, 2015	Dry cupping	Unclear	Back shu points	Acupuncture	16/8 weeks	2 per week/5 minutes
Li, 2012	Dry cupping	1-2	GB14 or EX-HN5	Acupuncture	16/15 days	Once a day/unclear
Zhang, 2020	Wet cupping	5	GV14, EX-HN5, TE5	Rizatriptan benzoate	4/20 days	1 per 5 days/5 minutes
Ersoy, 2020	Wet cupping	5	GV14, BL41-42, BL44-46	None	6 months	Demand rather than a single-month application/15 minutes

(1) 梅花穴: the edges and the central points of the pain area. (2) 項棱穴: 1.5 B-cun lateral to the cervical vertebra.

**Table 3:** Summary of findings and quality of the evidence.

Outcomes	Anticipated absolute effects (95% CI)		Relative effect (95% CI)	Number of participants (studies)	Certainty of the evidence (GRADE)
	Comparison	Intervention			
1. Wet-cupping compared to drugs for migraine					
1–1. Wet cupping compared to flunarizine for migraine					
TER	800 per 1,000	968 per 1,000 (800 to 1,000)	RR 1.21 (1.00 to 1.46)	60 (1 RCT)	⊕⊕○○ Low^a,b^

1–2. Wet cupping compared to flunarizine plus ibuprofen at acute attack for migraine					
TER	733 per 1,000	953 per 1,000 (792 to 1,000)	RR 1.30 (1.08 to 1.57)	90 (1 RCT)	⊕⊕○○ Low^a,c^
HI assessed with: VAS scale from 0 to 10	—	MD 1.4 lower (2.08 lower to 0.72 lower)	—	90 (1 RCT)	⊕⊕○○ Low^a,c^
Migraine score	—	MD 5.87 lower (7.66 lower to 4.08 lower)	—	90 (1 RCT)	⊕⊕○○ Low^a,c^

1–3. Wet cupping compared to diclofenac sodium at acute attack for migraine					
TER	800 per 1,000	968 per 1,000 (800 to 1,000)	RR 1.21 (1.00 to 1.46)	60 (1 RCT)	⊕⊕○○ Low^a,b^
HI assessed with VAS scale from 0 to 10	—	MD 0.35 lower (1.06 lower to 0.36 higher)	—	60 (1 RCT)	⊕⊕○○ Low^a,d^

2. Dry cupping plus acupuncture compared to acupuncture for migraine					
TER	941 per 1,000	988 per 1,000 (932 to 1,000)	RR 1.24 (0.99 to 1.12)	170 (2 RCTs)	⊕⊕○○ Low^a,b^
HI assessed with VAS scale from 0 to 10	—	MD 2.29 lower (2.62 lower to 1.96 lower)	—	70 (1 RCT)	⊕⊕○○ Low^a,c^
ND	—	MD 1.98 lower (2.91 lower to 1.05 lower)	—	100 (1 RCT)	⊕⊕○○ Low^a,c^

Explanations					
(a) Study had unclear risk of bias					
(b) Small sample size and the CI crosses 1					
(c) Small sample size					
(d) Small sample size and the CI crosses 0					
GRADE Working Group grades of evidence					
Moderate certainty: we are moderately confident in the effect estimate. The true effect is likely to be close to the estimate of the effect, but there is a possibility that it is substantially different.					
Low certainty: our confidence in the effect estimate is limited. The true effect may be substantially different from the estimate of the effect.					
Very low certainty: we have very little confidence in the effect estimate. The true effect is likely to be substantially different from the estimate of effect.					

CI = confidence interval, HI = headache intensity, MD mean difference, ND the number of days the headache has completely disappeared, RCT randomized controlled clinical trial, RR risk ratio, SMD standard mean difference, TER total effective rate, and VAS visual analogue scale.

## Data Availability

The data used to support the findings of this study are available from the corresponding author upon request.

## Abbreviations

AE: Adverse event
CI: Confidence interval
GRADE: Grading of recommendations assessment development and evaluation
HD: Headache duration time
HI: Headache intensity
MAS: Migraine-accompanying symptoms
MD: Mean difference
MF: Migraine attack frequency
MI: Migraine attack intensity
ND: The number of days the headache has completely disappeared
PRISMA: Preferred reporting items for systematic reviews and meta-analyses
RCT: Randomized controlled clinical trial
RR: Relative risk
SMD: Standard mean difference
TER: Total treatment effective rate
VAS: Visual analogue scale
24hMQOLQ: 24-hour migraine quality of life questionnaire.

## Supplementary Materials

Supplementary Materials Search strategies.
