# Supplementary material for: Cupping Therapy for Migraine: A PRISMA-Compliant Systematic Review and Meta-Analysis of Randomized Controlled Trials
Source: Evid Based Complement Alternat Med. 2021 Mar 24;2021:7582581. doi: 10.1155/2021/7582581 (PMC8016589; doi:10.1155/2021/7582581)
Supplement: Supplementary Materials — Search strategies. [file 7582581.f1.pdf]

Supplementary 1. Search strategies

Database	Search terms
MEDLINE	1. "Migraine Disorders"[Mesh]<br>2. "migraine disorders"[Title/Abstract]<br>3. "migraine attack"[Title/Abstract]<br>4. "episodic migraine"<br>5. headache[Title/Abstract]<br>6. (headache$[Title/Abstract] OR migrain$[Title/Abstract] OR cephalgi$[Title/Abstract] OR cephalalgi$[Title/Abstract])<br>7. (#1 OR #2 OR #3 OR #4 OR #5 OR #6)<br>8. Bloodletting[MeSH Terms]<br>9. "bloodletting therapy"[Title/Abstract]<br>10. cupping[Title/Abstract]<br>11. "wet cupping"[Title/Abstract]<br>12. "Blood-letting cupping"[Title/Abstract]<br>13. "venesection"[Title/Abstract]<br>14. phlebotomy[Title/Abstract]<br>15. "pricking therapy??Title/Abstract]<br>16. "pricking blood" [Title/Abstract]<br>17. "blood-draining" [Title/Abstract]<br>18. #8 OR #9 OR #10 OR #11 OR #12 OR #13 OR #14 OR #15 OR #16 OR #17<br>19. #7 AND #18
EMBASE	#1 'headache'/exp OR 'headache' OR 'migraine'/exp OR 'migraine' OR 'headache':ab,ti OR 'migraine':ab,ti OR headache*:ab,ti OR migrain*:ab,ti OR cephalgi*:ab,ti OR cephalalgi*:ab,ti OR 'migraine attack':ab,ti OR 'episodic migraine':ab,ti<br>#2 'venesection'/exp OR 'venesection' OR 'cupping'/exp OR 'cupping' OR 'cupping therapy'/exp OR 'cupping therapy' OR 'bloodletting therapy' OR 'bloodletting'/exp OR 'bloodletting'<br>#3 #1 AND #2<br>#4 #1 AND #2 AND [randomized controlled trial]/lim AND [humans]/lim
CENTRAL	#1 MeSH descriptor: [Migraine Disorders] explode all trees<br>#2 MeSH descriptor: [Headache] explode all trees<br>#3 (headache* or migrain* or cephalgi* or cephalalgi*):ti,ab,kw (Word variations have been searched)<br>#4 "migraine attack"<br>#5 "episodic migraine"<br>#6 #1 or #2 or #3 or #4 or #5<br>#7 MeSH descriptor: [Bloodletting] explode all trees<br>#8 ("bloodletting therapy"):ti,ab,kw (Word variations have been searched)<br>#9 (cupping):ti,ab,kw (Word variations have been searched)<br>#10 ("blood-letting cupping"):ti,ab,kw (Word variations have been searched)<br>#11 (venesection):ti,ab,kw (Word variations have been searched)<br>#12 #7 or #8 or #9 or #10 or #11<br>#13 #6 and #12
CNKI	1. 偏头痛 OR 发作性偏头痛 OR 偏头痛急性发作 OR 头静脉 OR Migraine<br>2. 拔罐 OR 火罐 OR 走罐 OR Cupping<br>3. #1 AND #2 ('结果中检索' was used)

CENTRAL = Cochrane Central Register of Controlled Trials, CNKI = China National Knowledge Infrastructure.
